# Wear-Resistant Carbon Nanotube-Reinforced Copper Nanocomposites: Microstructural Evolution and Tribological Performance

**DOI:** 10.3390/nano16211420

**Published:** 2026-11-04

**Authors:** Sónia Simões, Beatriz S. Monteiro, Alexandra C. Alves

**Affiliations:** 1 Department of Mechanical Engineering, Faculty of Engineering, University of Porto, Rua Doutor Roberto Frias, 4200-465 Porto, Portugal; ssimoes@fe.up.pt (S.S.); up201806755@edu.fe.up.pt (B.S.M.); 2 Associated Laboratory for Energy, Transports and Aerospace (LAETA), Institute of Science and Innovation in Mechanical and Industrial Engineering (INEGI), Rua Doutor Roberto Frias, 4200-465 Porto, Portugal

**Keywords:** copper nanocomposites, carbon nanotubes, powder metallurgy, wear resistance, tribological behaviour, electrical conductivity, microstructure-property relationships, EBSD

## Abstract

Carbon nanotube (CNT)-reinforced copper nanocomposites have attracted significant interest as multifunctional materials combining high electrical conductivity with enhanced wear resistance. In this work, Cu-CNT nanocomposites were fabricated using a powder metallurgy route and systematically investigated to establish the relationship between microstructure, tribological behaviour, and electrical performance. The materials were characterized by scanning electron microscopy (SEM), energy-dispersive spectroscopy (EDS), X-ray diffraction (XRD), and electron backscatter diffraction (EBSD) to evaluate CNT distribution and its influence on the copper matrix. Wear tests were performed to assess the functional performance of the composites. The results show that CNT incorporation significantly improved the wear resistance of copper, leading to reduced material loss and modifications in the dominant wear mechanisms. The enhanced tribological behaviour was associated with the reinforcing effect of CNTs, improved load transfer, and surface protection during sliding. Microstructural analyses revealed that CNT addition influenced the evolution of the copper matrix, contributing to improved durability. Importantly, the composites retained high electrical conductivity despite the presence of the nanoscale reinforcement. These results demonstrate the potential of Cu-CNT nanocomposites for conductive components operating under wear conditions and provide further insight into the microstructure-property relationships governing their multifunctional performance.

## 1. Introduction

Copper (Cu) is one of the most important engineering materials for electrical, electronic, and thermal management applications, owing to its high electrical and thermal conductivity, good corrosion resistance, and excellent formability. However, its relatively low hardness and limited wear resistance restrict its use in components subjected to severe tribological conditions. Consequently, the development of Cu-based nanocomposites that exhibit a combination of high electrical conductivity, improved mechanical properties and enhanced tribological performance represents a solution for expanding the potential applicability of Cu-based materials [1,2,3].

Among the various reinforcements employed in metal matrix composites, carbon nanotubes (CNTs) have emerged as particularly attractive candidates because of their exceptional tensile strength, high Young’s modulus, low density, and outstanding electrical and thermal conductivity [4]. The incorporation of CNTs into copper matrices has the potential to simultaneously improve mechanical, tribological, and functional properties, making Cu-CNT composites promising materials for applications such as electrical contacts, electrodes, heat sinks, sliding conductive components, and next-generation electronic devices [1,2,3].

The superior performance of CNT-reinforced copper composites is generally attributed to several strengthening mechanisms, including load transfer from the matrix to the nanotubes, grain refinement, Orowan strengthening, and the accumulation of geometrically necessary dislocations around the reinforcement phase [5,6]. Furthermore, CNTs can effectively hinder grain boundary migration during consolidation processes, resulting in finer microstructures and consequently increased hardness and strength [6]. In addition, the intrinsic lubricating characteristics of carbon nanotubes can contribute to reducing friction and minimizing material removal during sliding contact, thereby improving wear resistance [7,8].

Besides mechanical and tribological enhancements, considerable attention has been devoted to the electrical performance of Cu-CNT composites. In principle, the combination of highly conductive copper and CNTs should enable the development of advanced conductive materials with superior multifunctional properties [3]. Nevertheless, the conductivity achieved experimentally often falls below theoretical predictions because it is strongly influenced by CNT dispersion, nanotube alignment, interfacial bonding, porosity, and processing-induced defects [1,3]. Poor interfacial compatibility between copper and carbon, together with the tendency of CNTs to agglomerate due to strong van der Waals interactions, frequently results in heterogeneous microstructures that increase electron scattering and impair charge transport through the composite [1,2,3,9,10]. Therefore, achieving homogeneous CNT dispersion and strong matrix–reinforcement interfaces remains one of the principal challenges in the fabrication of high-performance Cu-CNT composites [1,9,10].

Recent studies have demonstrated that CNT addition can significantly increase hardness, strength, and wear resistance; however, the resulting electrical conductivity strongly depends on the reinforcement content and microstructural characteristics of the composite [2,7,10,11,12,13]. While low CNT contents may provide effective strengthening with limited impact on conductivity, excessive additions often promote CNT clustering, porosity formation, and increased interfacial resistance, ultimately reducing electrical performance [1,2,7,9,10]. Consequently, establishing an optimal CNT concentration capable of maximizing wear resistance while preserving the excellent conductivity of copper remains a critical issue for the development of multifunctional Cu-based nanocomposites.

Although considerable research has been devoted to Cu-CNT composites, most studies have primarily focused on mechanical strength and hardness, whereas fewer investigations have systematically correlated CNT content with wear behaviour, electrical conductivity, and detailed microstructural features over a broad reinforcement range [1,2,5,10,11,12,13]. Furthermore, the influence of CNT additions above 2 wt.% on grain structure evolution, crystallographic characteristics, and the resulting wear–conductivity trade-off remains insufficiently understood. Therefore, a comprehensive microstructural and functional assessment is required to establish the optimal CNT content for advanced multifunctional Cu-CNT nanocomposites [1,2,5,7,10].

Microstructural characterization plays a fundamental role in understanding the mechanisms controlling the performance of Cu-CNT composites. Scanning electron microscopy (SEM) enables the analysis of CNT distribution, porosity, wear mechanisms, and fracture features, while electron backscatter diffraction (EBSD) provides detailed information regarding grain size, grain boundary characteristics, recrystallization phenomena, local misorientations, and crystallographic texture evolution [11]. Combined with hardness measurements, these techniques allow the establishment of direct relationships between microstructural evolution and the resulting mechanical, tribological, and electrical properties.

Therefore, the present work investigates Cu-CNT nanocomposites containing CNT contents ranging from 0.5 to 2.5 wt.%. The influence of reinforcement content on microstructure, hardness, wear resistance, and electrical conductivity was systematically evaluated. SEM and EBSD analyses were performed to characterize the microstructural evolution induced by CNT addition and to identify the underlying strengthening mechanisms. Attention was given to understanding the relationship between CNT content, grain structure, tribological behaviour, and electrical performance to determine the reinforcement level that provides the most favourable balance between wear resistance and electrical conductivity.

## 2. Materials and Methods

### 2.1. Raw Materials

Commercially pure copper (Cu) powder (Goodfellow Cambridge Ltd., Huntingdon, UK) was used as the matrix material, while multi-walled carbon nanotubes (MWCNTs), supplied by Fibermax (Fibermax Ltd., London, UK), were employed as the reinforcement phase. MWCNTs were used as reinforcement over single-walled carbon nanotubes (SWCNTs) due to their high aspect ratio, mechanical robustness, and electrical properties, which make them suitable for reinforcement in metal-matrix composites. The employed MWCNTs had an average number of walls of 17 ± 6, an outer diameter of 18.7 ± 6.5 nm, an inner diameter of 5.0 ± 1.0 nm, and a length ranging from 1 to 25 μm, as specified in a previous work [14]. Cu-CNT nanocomposites containing 1.0, 1.5, 2.0 and 2.5 wt.% CNTs were produced to evaluate the influence of reinforcement content on the microstructure, hardness, wear resistance, and electrical conductivity.

### 2.2. Preparation of Cu-CNT Nanocomposite Powders

The Cu-CNT nanocomposite powders were produced by powder metallurgy using an ultrasonication-assisted dispersion route following methodologies previously developed and optimized by Carneiro et al. and Monteiro et al. [15,16,17]. These studies demonstrated that ultrasonication is an effective technique for promoting CNT deagglomeration while minimizing structural damage and improving reinforcement distribution throughout the metallic matrix.

Initially, the required amount of CNTs was dispersed in isopropyl alcohol. The CNT suspension was subjected to ultrasonication for 15 min to promote nanotube deagglomeration and homogeneous dispersion. Subsequently, the copper powder was added to the suspension and dispersion/mixing was continued under ultrasonic agitation, allowing CNTs to become attached to the surface of the Cu particles. The use of a liquid medium during ultrasonication reduces van der Waals interactions between nanotubes and promotes a more uniform distribution of the reinforcement phase throughout the powder mixture [15,16,17].

After ultrasonication, the suspension was dried until complete solvent evaporation. The dried powder mixtures were then gently disaggregated and filtered to obtain a homogeneous feedstock for compaction.

The powder metallurgy route employed in the present study has potential for scale-up, as powder metallurgy is an established manufacturing route for Cu-based composites [18,19]. However, this may require particular attention regarding CNT dispersion in larger powder batches, as inadequate dispersion may promote nanotube agglomeration, non-uniform reinforcement distribution and unwanted porosity. Potential approaches to address this challenge can include the optimisation of ultrasonication conditions and the usage of scalable mixing technologies.

### 2.3. Compaction and Sintering

The Cu-CNT powder mixtures were consolidated through uniaxial cold pressing followed by sintering. The powders were compacted at 900 MPa using a hardened steel die to produce cylindrical specimens.

The green compacts were subsequently sintered under high vacuum to promote densification while minimizing copper oxidation for 120 min at 950 °C. The sintering temperature and holding time were selected to achieve adequate diffusion bonding between particles while preserving the integrity of the CNT reinforcement.

To enable a direct comparison between different CNT contents, all compositions were processed under identical compaction and sintering conditions.

### 2.4. Microstructural Characterization

The microstructure of the sintered Cu-CNT nanocomposites was characterized through scanning electron microscopy (SEM), using a Phenom XL G2 Desktop SEM (Thermo Fisher Scientific, Hillsboro, OR, USA). Prior to observation, the specimens were sectioned, mounted, ground with successive SiC abrasive papers, and subsequently polished using diamond suspensions until a mirror-like surface finish was obtained. SEM (Thermo Fisher Scientific QUANTA 400 FEG-ESEM, Thermo Fisher Scientific, Hillsboro, OR, USA). analyses were performed to evaluate the microstructural homogeneity of the composites, the distribution and dispersion of CNTs within the copper matrix, particle bonding, residual porosity, and the degree of densification achieved after sintering. Furthermore, the worn surfaces and wear tracks generated during tribological testing were examined to identify the dominant wear mechanisms and correlate them with the reinforcement content.

Electron backscatter diffraction (EBSD) (TSL-EDAX EBSD Unit, EDAX Inc. (Ametek), Mahwah, NJ, USA) was employed to investigate the crystallographic characteristics and grain structure of the composites. The analyses were performed on mechanically polished specimens, and the acquired data were processed according to procedures previously reported for powder metallurgy metal matrix nanocomposites by Carneiro et al. and Simões et al. [14,15]. EBSD characterization included the acquisition of inverse pole figure (IPF) maps for crystallographic orientation analysis, grain size determination, grain boundary character distribution, Kernel Average Misorientation (KAM) maps, and crystallographic texture evaluation. Grain size measurements were obtained from the EBSD grain maps using the equivalent circle diameter approach. Boundaries exhibiting misorientation angles above 15° were classified as high-angle grain boundaries (HAGBs), while boundaries with lower misorientation values were considered low-angle grain boundaries (LAGBs). The EBSD analysis was further used to assess the influence of CNT content on grain refinement, texture development, and local lattice distortions, providing a better understanding of the microstructural mechanisms governing the hardness, wear resistance, and electrical conductivity of the Cu-CNT nanocomposites.

### 2.5. Hardness Measurements

The hardness of the composites was determined by Vickers microhardness testing (HV0.01) using a load of 98 mN, for a normalized indentation time of 15 s, using a Duramin-1 durometer (Struers A/S, Ballerup, Denmark). Measurements were carried out on polished cross-sections under a constant load and dwell time.

For each condition, at least 12 indentations were performed at different locations to ensure statistical significance. The reported hardness values correspond to the average value and standard deviation, which were used to assess the strengthening effect associated with CNT addition and the microstructural modifications promoted by the reinforcement.

### 2.6. Wear Tests

The wear performance of the nanocomposites was investigated under dry reciprocating sliding conditions using a CETR-UMT2 tribometer (Bruker, Campbell, CA, USA) in a ball-on-disk configuration. Alumina (Al_2_O_3_) balls with a diameter of 10 mm, supplied by Goodfellow (Goodfellow Cambridge Ltd., Huntingdon, UK), were employed as the counter-body. The tests were conducted under a constant normal load of 5 N, with at least three repetitions per three independent samples, performed to assess the repeatability of the measurements. A frequency of 1 Hz and a total stroke length of 5 mm were applied for a sliding duration of 15 min at room temperature. Following testing, the wear tracks and the alumina counter-bodies were analysed by SEM and EDS to characterize the resulting wear features and determine the predominant wear mechanisms.

### 2.7. Resistivity Measurements

The electrical resistivity measurements were performed using the four-point probe method [20], by resorting to an Ossila Four-Point Probe equipment (Ossila Ltd., Sheffield, UK), at room temperature.

## 3. Results and Discussion

### 3.1. Characterization of the As-Received Powders

**Figure 1:**
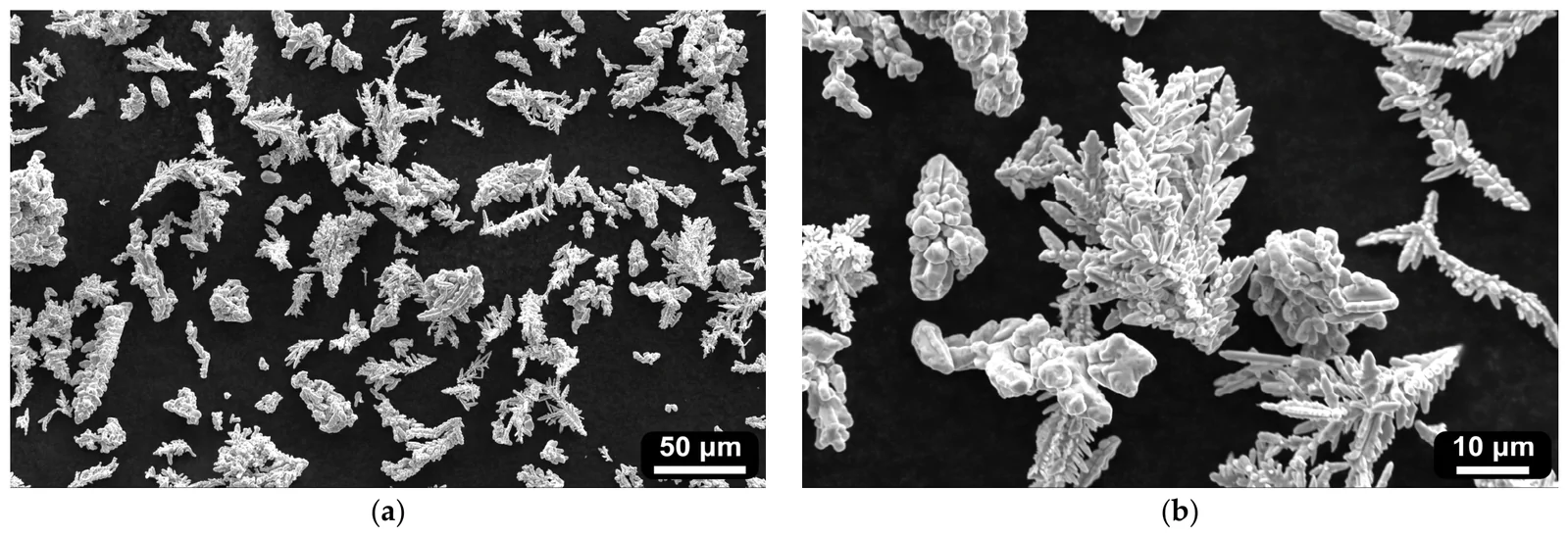
Characterization of the copper powder used as matrix material: (**a**) low-magnification SEM image, (**b**) high-magnification SEM image showing the dendritic morphology of the particles.

**Figure 2:**
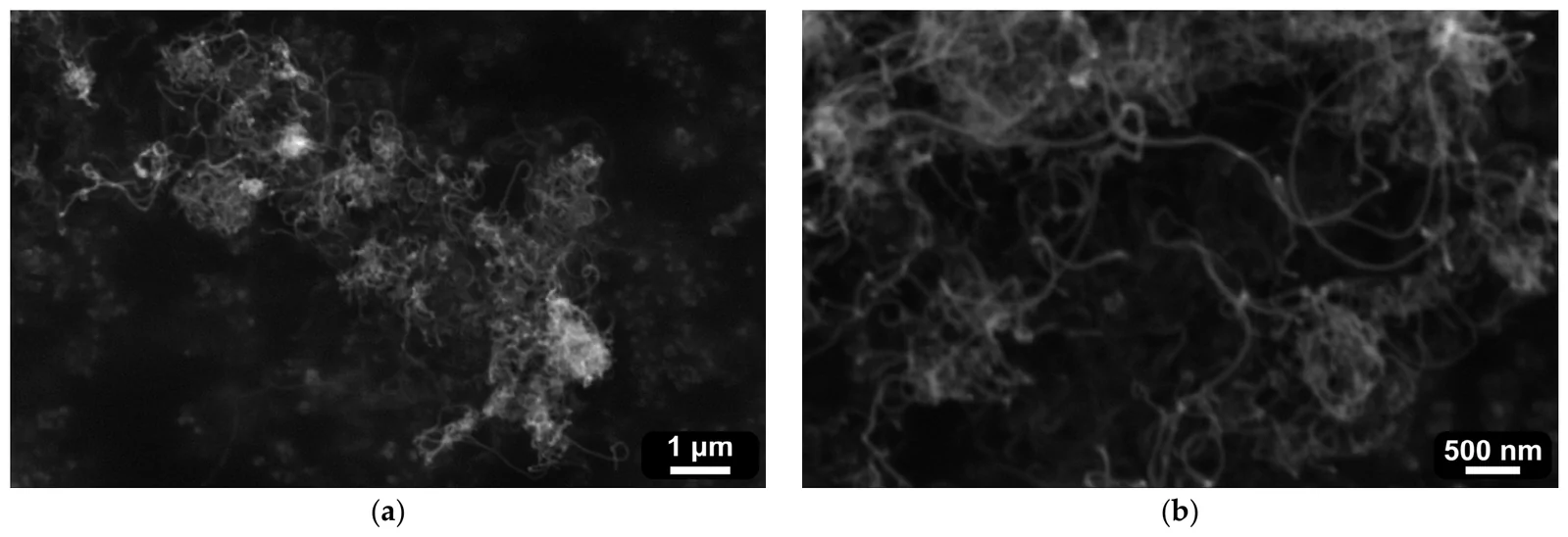
Multi-walled carbon nanotubes (MWCNTs) used as reinforcement: (**a**) low-magnification and (**b**) high-magnification SEM images showing their characteristic entangled network morphology and high aspect ratio.

Figure 1 presents representative SEM micrographs and the particle size distribution of the as-received copper powder; according to the DLS analysis reported previously [15], the powder exhibited a D_50_ of 29.45 μm. As shown in Figure 1, the particles are characterized by an elongated morphology, resulting in substantial differences between their length and width. Therefore, the D_50_ value is reported as a representative measure of particle size, while the particle geometry is more accurately described by the observed morphology. On the other hand, Figure 2 shows the morphology of the multi-walled carbon nanotubes (MWCNTs) used as reinforcement. The characterization of the starting materials is essential to assess their suitability for powder metallurgy processing and their potential influence on CNT dispersion and composite densification. The SEM micrographs revealed that the copper powders exhibited a predominantly dendritic morphology, characterized by irregular and elongated particles with a rough surface texture. Such morphology is commonly observed in atomized copper powders and can be advantageous for mechanical interlocking during compaction, promoting adequate green strength before sintering. The irregular surface features may also facilitate the attachment of CNTs during the ultrasonication-assisted dispersion process, contributing to a more homogeneous distribution of the reinforcement throughout the powder mixture. The particle size distribution analysis indicated an average particle size in the micrometric range, with a relatively broad distribution typical of commercial copper powders. The mean particle size was sufficiently small to provide a high surface area for CNT attachment while remaining large enough to ensure good powder flowability and handling during processing. Such characteristics are expected to favour effective densification during sintering and contribute to the development of a homogeneous microstructure in the consolidated composites. The CNTs exhibited the characteristic tubular morphology and high aspect ratio expected for multi-walled carbon nanotubes. Their elongated geometry provides a large interfacial area for interaction with the copper matrix, which may be beneficial for improving the functional properties of the composites. However, owing to strong van der Waals interactions between nanotubes, achieving a homogeneous dispersion remains a critical challenge. Therefore, the characteristics of both the copper powders and the CNTs highlight the importance of the selected dispersion route to promote uniform reinforcement distribution while minimizing CNT agglomeration. Overall, the morphology and particle size of the copper powders, together with the structural characteristics of the CNTs, indicate that the selected raw materials are suitable to produce Cu-CNT nanocomposites by powder metallurgy and provide favourable conditions for subsequent mixing, compaction, and sintering processes.

### 3.2. Microstructure of the Sintered Cu-CNT Nanocomposites

The interfacial interaction between CNTs and the Cu matrix is strongly influenced by the surface chemistry of the carbon nanotubes. Owing to their graphitic structure, CNTs are intrinsically chemically inert and exhibit limited wettability by molten or solid-state copper, which may result in weak interfacial bonding. Surface defects and oxygen-containing functional groups can enhance the affinity between CNTs and the Cu matrix, promoting improved dispersion and more effective load transfer across the interface. Consequently, the nature of the CNT/Cu interface plays a critical role in determining the mechanical and tribological performance of Cu-CNT composites. In the present work, no additional chemical functionalization of the CNTs was performed. Therefore, the interfacial interaction is expected to arise primarily from physical contact and mechanical interlocking developed during powder processing and consolidation.

**Figure 3:**
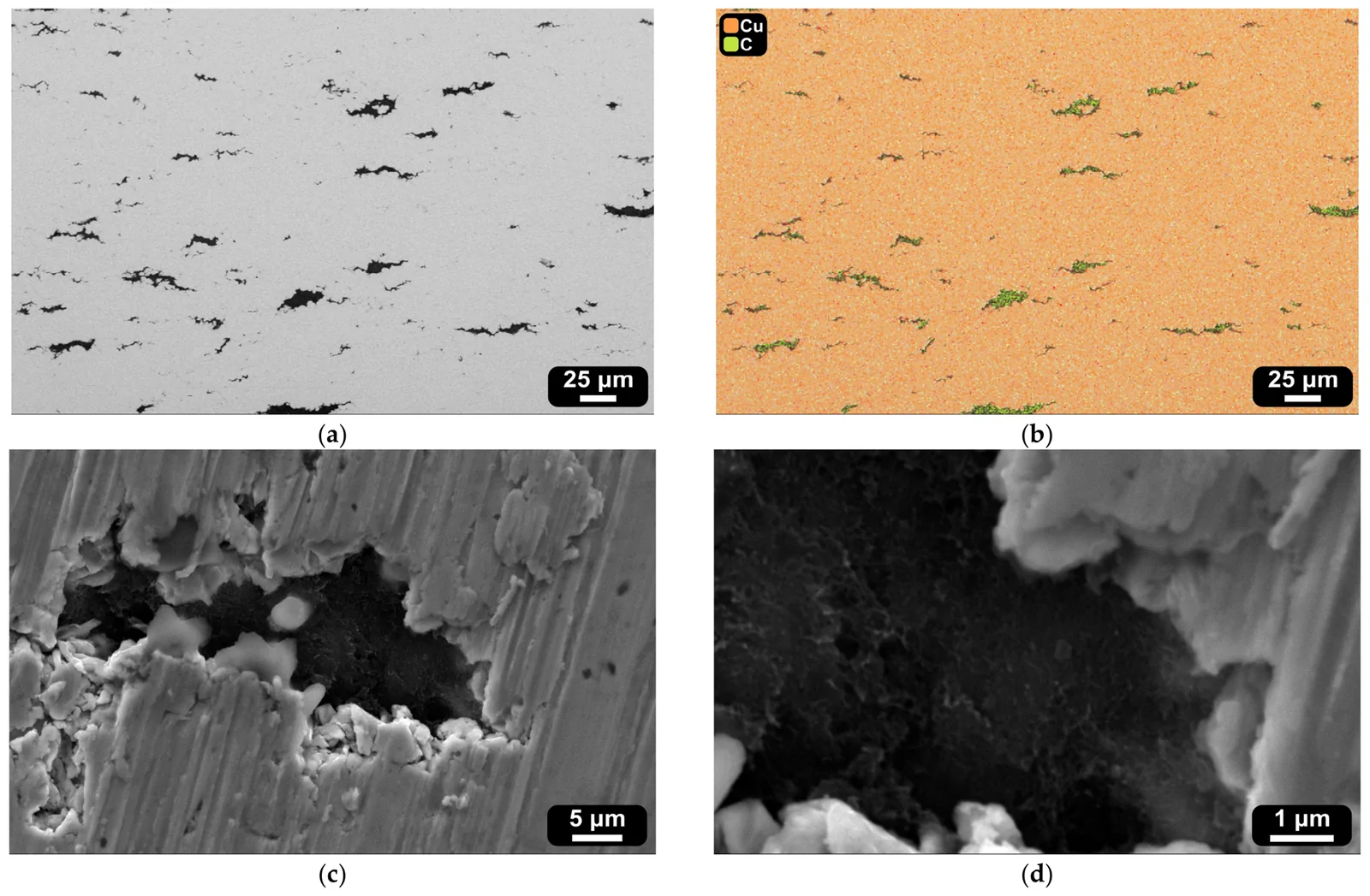
Microstructural characterization of sintered Cu-1CNT nanocomposites: (**a**) SEM micrograph of a polished cross-section; (**b**) corresponding elemental distribution map showing the Cu and C distributions; (**c**) SEM micrograph revealing a pore containing a CNT agglomerate; and (**d**) higher-magnification image showing the entangled CNT network within the agglomerated region.

The detailed microstructural characterization of Cu-CNT nanocomposites produced through the same powder metallurgy route, including grain structure evolution, CNT distribution, densification behaviour, hardness, tensile properties, and strengthening mechanisms, has been reported [15,16,17]. Previous studies demonstrated that the incorporation of CNTs can improve the mechanical performance of the composites when an adequate dispersion of the reinforcement is achieved [15,16,17]. Therefore, the present section focuses exclusively on the microstructural features that are directly relevant to the thermal conductivity and tribological behaviour investigated in this work. Representative SEM images, elemental distribution maps, and high-magnification micrographs of polished cross-sections of the sintered Cu-CNT nanocomposites are presented in Figure 3, while the evolution of hardness and the combined area fraction of pores and CNT agglomerates is summarized in Table 1. The SEM micrographs reveal the presence of elongated dark phase distributed throughout the Cu matrix, which correspond to carbon-rich regions. This is confirmed by the elemental maps, where the carbon signal is localized in the same areas. Higher-magnification observations further show that these regions consist of CNT agglomerates, frequently associated with pores or interparticle boundaries, indicating that complete CNT dispersion was not achieved during powder processing. Such agglomerates contribute to the measured defect area fraction and may locally influence densification and mechanical response.

The SEM observations revealed a clear dependence of the microstructural homogeneity on CNT content. For the composite containing 1 wt.% CNT, the reinforcement was relatively well distributed throughout the copper matrix, with limited evidence of large, agglomerated regions. This homogeneous distribution is consistent with the higher hardness values obtained for this composition (83 ± 6 HV0.01), suggesting that the dispersion procedure was effective in promoting an adequate interaction between the reinforcement and the matrix.

As the CNT content increased above 1 wt.%, the microstructure became progressively more heterogeneous. CNT-rich regions, clusters, and isolated agglomerates were increasingly observed throughout the matrix, accompanied by a gradual increase in the fraction of porosity and microstructural defects. Quantitative image analysis showed that the combined area fraction of pores and CNT agglomerates increased from 1.95% for the Cu-1CNT composite to 6.29% for the Cu-2.5CNT material. This behaviour is attributed to the increasing difficulty of overcoming the strong van der Waals interactions between nanotubes as the reinforcement content increases, resulting in localized CNT accumulation and less efficient densification during sintering.

Mallikarjuna et al. [21] reported that the absence of significant porosity and the uniform distribution of the reinforcements contribute to improved interfacial bonding between the Cu matrix and the reinforcing phase. In contrast, reinforcement agglomeration can promote pore formation, leading to a reduction in densification and creating regions with weaker matrix–reinforcement adhesion. Therefore, a homogeneous dispersion of the reinforcements, together with high density values, suggests that the reinforcing particles are well distributed throughout the matrix and effectively bonded to the surrounding Cu. Furthermore, the presence of uniformly dispersed reinforcements provides an effective barrier to localized plastic deformation during indentation, thereby contributing to the enhancement of the composite hardness.

The evolution of hardness further supports the observed microstructural changes. While the Cu-1 wt.% CNT composite exhibited the highest hardness, a progressive decrease was observed for higher CNT concentrations, reaching 52 ± 5 HV0.01 for the Cu-2.5 wt.% CNT sample. This reduction is likely associated with the increasing amount of porosity and reinforcement agglomeration, which counteracts the potential benefits of the CNT addition and reduces the effectiveness of load transfer within the composite.

Although the strengthening mechanisms associated with CNT incorporation have been extensively addressed in previous studies [16], the present results highlight the dominant role of microstructural homogeneity in controlling the functional performance of the composites. In particular, the degree of CNT dispersion and the amount of residual porosity are expected to significantly influence heat transport and wear resistance. A homogeneous microstructure provides more continuous conductive pathways and a more uniform stress distribution during sliding contact, whereas CNT agglomerates and pores act as preferential sites for phonon and electron scattering, crack initiation, and localized material removal.

Consequently, the microstructural observations suggest the existence of an optimum CNT content at which reinforcement dispersion is maximized while defect formation remains limited. This balance is expected to be reflected in the electrical conductivity and tribological results discussed in the following sections.

### 3.3. X-Ray Diffraction Analysis

**Figure 4:**
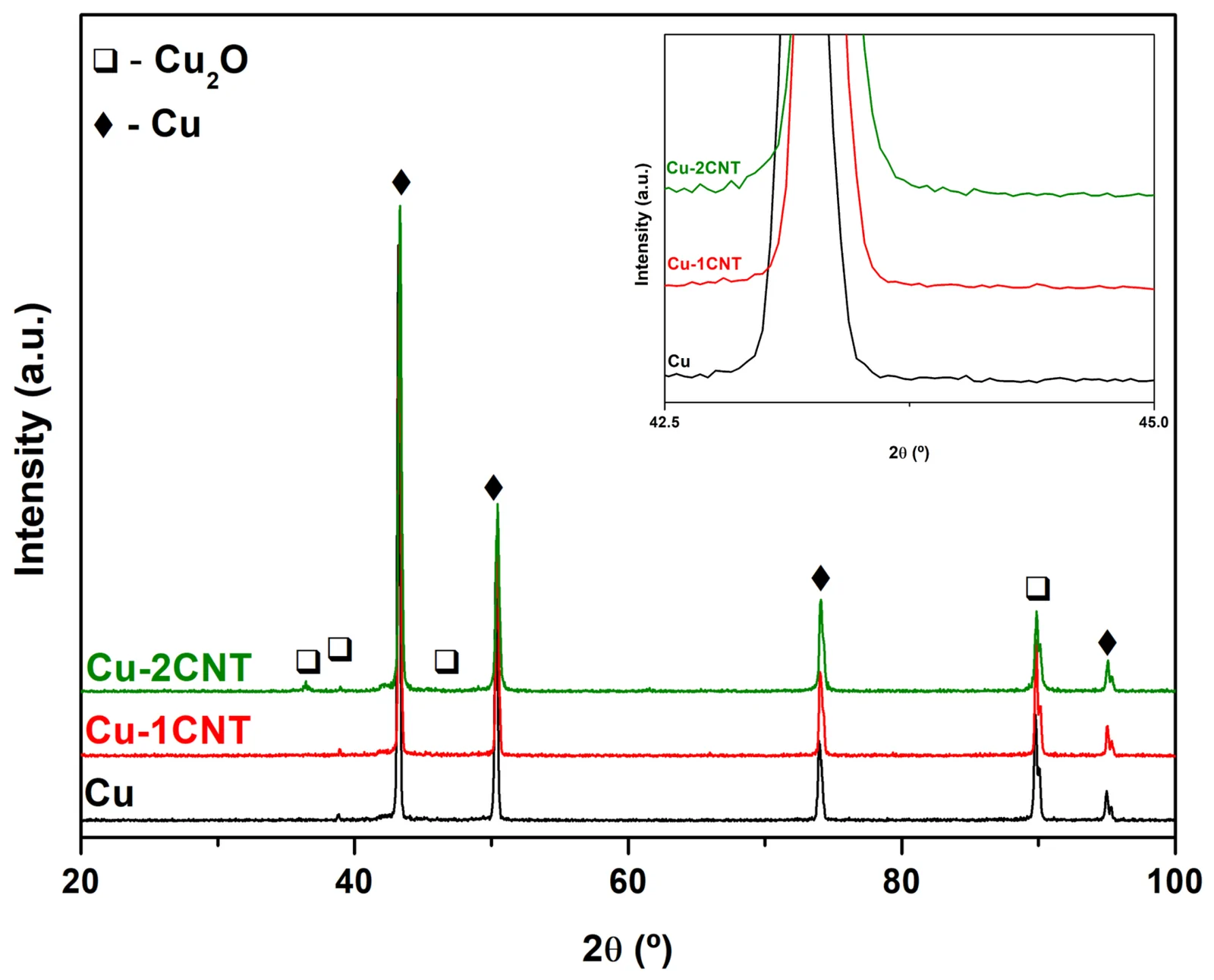
XRD patterns of pure Cu, Cu-1CNT and Cu-2CNT nanocomposites, together with the enlarged view of the Cu(111) diffraction peak.

The X-ray diffraction patterns of pure copper and Cu-CNT nanocomposites are presented in Figure 4, with a detailed view of the Cu(111) diffraction peak. The XRD analysis was performed to identify the phases present after sintering and to evaluate the influence of CNT incorporation on the crystallographic structure of the copper matrix.

The diffraction patterns of all materials were dominated by the characteristic reflections of face-centred cubic (FCC) copper, corresponding to the (111), (200), (220), and (222) crystallographic planes. These results indicate that the copper matrix retained its crystallographic structure after processing and sintering, independently of the CNT content.

Besides the Cu reflections, peaks associated with Cu_2_O were also detected in both unreinforced Cu and the Cu-CNT samples, indicating the presence of a limited amount of oxide formed during powder handling and consolidation. Nevertheless, the intensity of these oxide peaks remained relatively low compared with the main Cu reflections, confirming that the metallic phase remained predominant. More importantly, no diffraction peaks associated with copper carbides or other reaction products were identified, demonstrating that CNT incorporation did not promote significant chemical reactions with the copper matrix during processing.

The preservation of the Cu crystalline structure is particularly important for the functional performance of the composites. Since no undesirable secondary phases were formed, the intrinsic electrical conductivity of copper is expected to be largely preserved, and any variations in electrical transport should mainly originate from microstructural factors such as CNT dispersion, interfacial thermal resistance, and residual porosity. 2θ position together with Full Width at Half Maximum (FWHM) parameters are shown in Table 2.

A detailed analysis of the Cu(111) and Cu(200) reflections revealed minor shifts in peak position with increasing CNT content. The Cu(111) peak was located at 43.20°, 43.26°, and 43.31° for Cu, Cu-1CNT, and Cu-2CNT, respectively, while the Cu(200) reflection shifted slightly from 50.31° to 50.41°. These small variations suggest that CNT incorporation did not significantly alter the lattice parameter of copper and that no substantial solid-solution effects occurred.

Changes in peak broadening were also observed. In particular, the FWHM of the Cu(200) peak increased from 0.2362° for pure Cu to 0.3149° for the Cu-1CNT composite, while remaining higher than the unreinforced material for the higher CNT content. Such peak broadening may be associated with the introduction of local lattice distortions, microstrain, and structural heterogeneities resulting from the presence of CNT-matrix interfaces. Similar effects have been reported in metal matrix nanocomposites reinforced with carbon nanotubes, where the incorporation of nanoscale reinforcements generates additional crystallographic defects and local strain fields [5,6].

Overall, the XRD results confirm that the selected powder metallurgy route successfully incorporated CNTs without inducing the formation of undesirable reaction products. The absence of carbide phases and the preservation of the FCC copper structure indicate good chemical compatibility between the matrix and the reinforcement. Furthermore, the observed peak broadening suggests the development of local microstructural distortions, which may influence the wear behaviour discussed in the following sections.

### 3.4. Microstructural Evolution Assessed by EBSD

**Figure 5:**
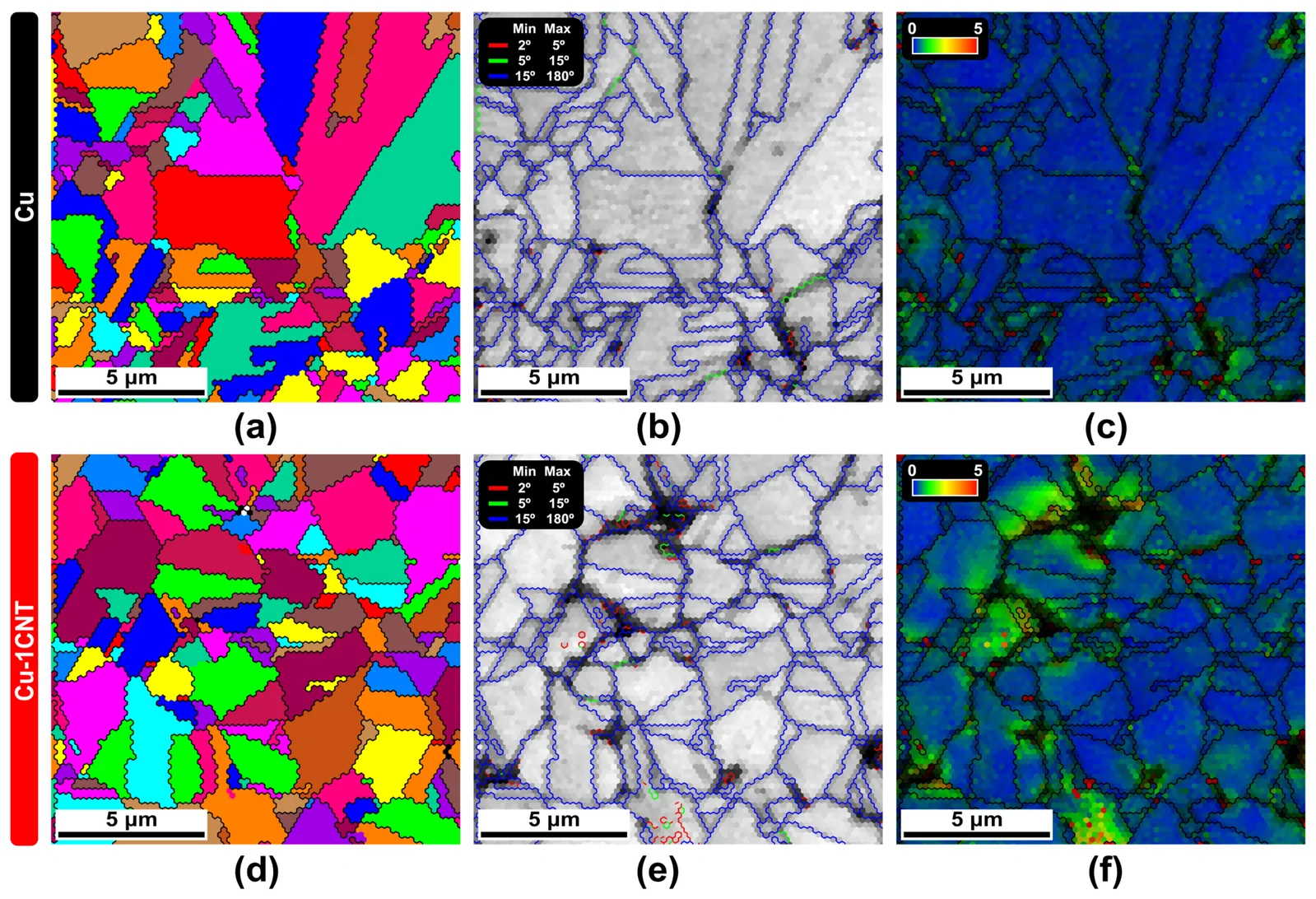
EBSD characterization of Cu and Cu-CNT nanocomposites: (**a**,**d**) unique color grain maps, (**b**,**e**) grain boundary maps, and (**c**,**f**) KAM maps for (**a**–**c**) unreinforced Cu and (**d**–**f**) Cu-1CNT.

**Figure 6:**
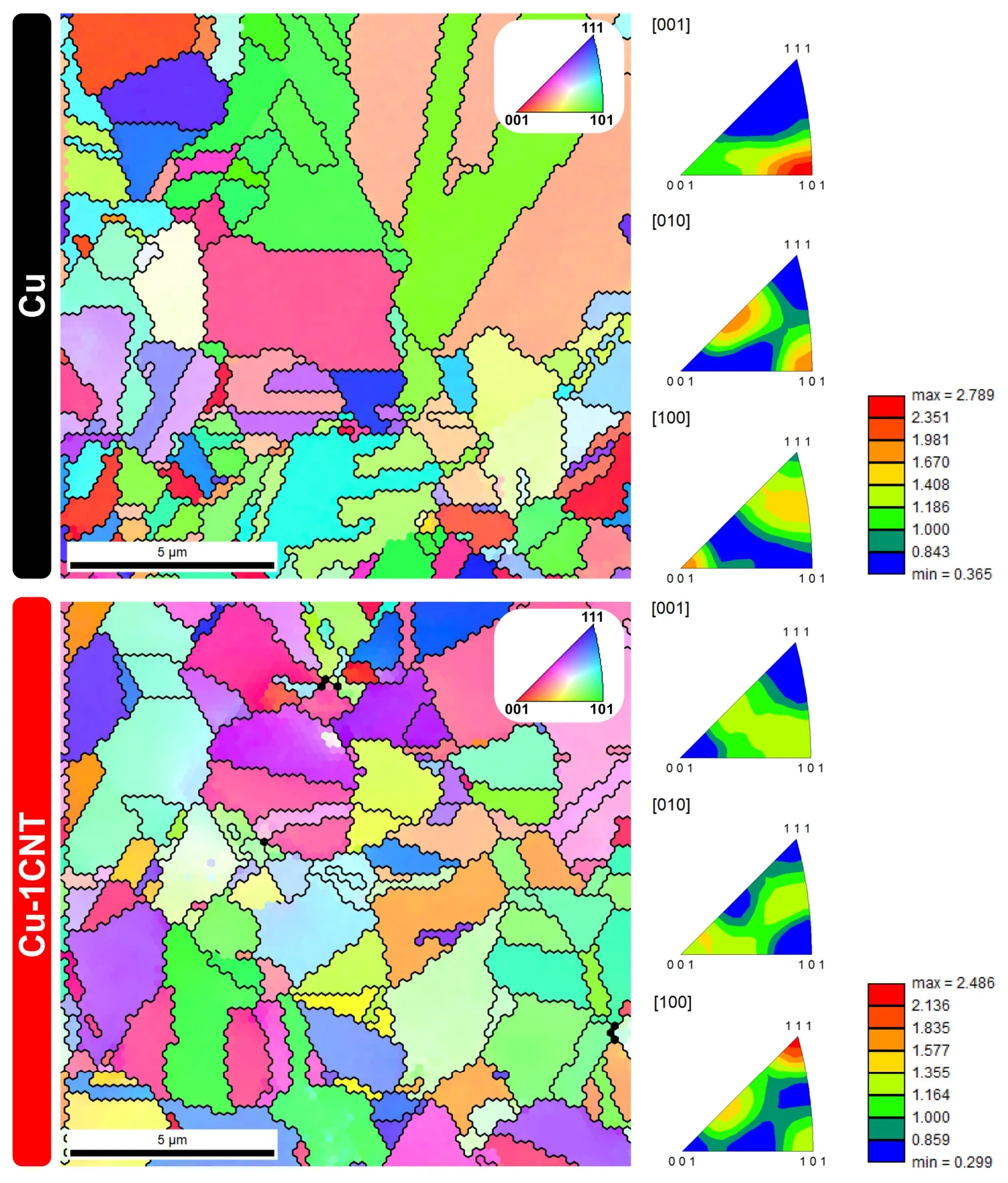
Inverse pole figures of Cu and Cu-1CNT nanocomposites.

The microstructural evolution of the Cu-CNT nanocomposites was further investigated by electron backscatter diffraction (EBSD). Since a detailed analysis of grain refinement mechanisms and their influence on the mechanical properties of these materials has been previously reported [15,16,17], the present section focuses on the microstructural features that are relevant for understanding the electrical conductivity and tribological performance of the composites. Representative grain size distributions, grain boundary maps and kernel average misorientation (KAM) maps are presented in Figure 5, while inverse pole figures are presented in Figure 6 for texture analysis. The corresponding quantitative EBSD parameters, including average grain size, fraction of low-angle grain boundaries (LAGBs), fraction of high-angle grain boundaries (HAGBs), and average local misorientation, are summarized in Table 3.

The unique colour maps revealed that both materials exhibit a microstructure composed of equiaxed grains. However, the incorporation of 1 wt.% CNT resulted in a slightly more homogeneous microstructure, characterized by a reduction in the fraction of coarse grains and a more uniform grain size distribution. In agreement with the quantitative EBSD measurements, the average grain size decreased from 1.34 ± 0.9 μm in pure Cu to 1.18 ± 0.7 μm in the Cu-1CNT composite. This behaviour suggests that CNTs partially restricted grain boundary migration during sintering, acting as effective pinning points and inhibiting grain growth.

The grain boundary analysis further supports this behaviour. The Cu-1CNT composite exhibited a slightly higher fraction of low-angle grain boundaries (LAGBs), increasing from 0.079 in pure Cu to 0.090 after CNT addition. High-angle grain boundaries (HAGBs) remained essentially unchanged, increasing only from 0.37 to 0.38. In contrast, the fraction of coincidence site lattice (CSL) boundaries decreased slightly from 0.56 to 0.53. These results suggest that CNT incorporation promoted localized strain accommodation during sintering, favouring the formation of subgrain structures and low-misorientation boundaries without substantially altering the overall grain boundary network.

This behaviour is consistent with the Kernel Average Misorientation (KAM) analysis. KAM maps provide information on the local crystallographic misorientation between neighbouring measurement points and are commonly used as an indicator of stored strain and lattice distortion. The average KAM value increased from 0.55° for pure Cu to 0.58° for the Cu-1CNT composite, indicating a slight increase in local lattice strain. The KAM maps revealed that regions with higher misorientation were preferentially located near grain boundaries and CNT-rich regions identified by SEM observations. This behaviour suggests that CNTs introduced local strain fields within the copper matrix, likely associated with thermal expansion mismatch, interfacial constraints, and the accumulation of geometrically necessary dislocations around the reinforcement.

Additional insight into the crystallographic orientation distribution was obtained from the Inverse Pole Figure (IPF) maps. IPF maps display the crystallographic orientation of individual grains and are widely used to assess texture development and orientation heterogeneity within the microstructure. The IPF maps revealed that both materials exhibited a relatively broad distribution of grain orientations, with no dominant crystallographic direction, indicating an overall weak texture. Nevertheless, the incorporation of 1 wt.% CNT induced subtle changes in the crystallographic texture. Although both materials showed dispersed orientation distributions, the maximum texture intensity decreased from 2.79 MUD for pure Cu to 2.49 MUD for Cu-1CNT. This reduction indicates a slight randomisation of crystallographic orientations and suggests that CNTs influenced the recrystallisation behaviour of the copper matrix. The nanotubes located at particle interfaces likely hindered preferential grain growth during consolidation, resulting in a more isotropic microstructure.

The EBSD results are in good agreement with the XRD analysis, which showed increased diffraction peak broadening in the Cu-CNT composites. The higher KAM values and the slight increase in LAGB fraction support the presence of additional local lattice distortions and microstrain generated by the CNT-matrix interfaces. These microstructural features are expected to contribute to the hardness improvement observed in the composites by increasing resistance to dislocation motion.

From a functional perspective, the relatively limited grain refinement observed suggests that grain size effects are not the dominant factor controlling the electrical and tribological behaviour of the materials. Instead, the increased density of interfaces, local strain fields, and CNT-rich regions is likely to play a more significant role. While these features can enhance mechanical strengthening and load-bearing capacity during sliding contact, they may also act as additional electron-scattering sites, contributing to the reduction in electrical conductivity observed with increasing reinforcement content.

Overall, the EBSD analysis indicates that the addition of 1 wt.% CNT induces moderate grain refinement and a slight increase in local lattice distortion without significantly modifying the overall grain boundary character of the copper matrix. The combination of a finer grain structure, increased fraction of low-angle grain boundaries, and higher local misorientation provides microstructural evidence for the strengthening mechanisms operating in the Cu-1CNT composite and helps explain the observed balance between hardness enhancement and electrical conductivity reduction.

### 3.5. Tribological Performance

The tribological test parameters were selected to ensure measurable wear while maintaining stable contact conditions and avoiding excessive damage to the samples. In particular, the normal load of 5 N was chosen as a compromise between generating sufficient wear for reliable characterization of wear mechanisms and minimizing the risk of severe material removal that could mask the influence of CNT reinforcement. Furthermore, loads in the range of 5–50 N have been widely employed in tribological studies of Cu-CNT composites [12,21,22,23,24,25,26]. Therefore, the selected load lies within the lower end of the range commonly reported in the literature, facilitating comparison with previous studies while providing reproducible wear and friction data. Hertzian contact calculations indicated that the selected 5 N load generated a maximum contact pressure of approximately 0.76 GPa. This pressure is within the range commonly employed in laboratory tribological studies and is sufficiently high to produce measurable wear while avoiding excessively severe contact conditions or premature surface failure. Consequently, the selected load enabled a reliable assessment of the influence of CNT incorporation on the tribological behavior of the Cu-CNT composites.

**Figure 7:**
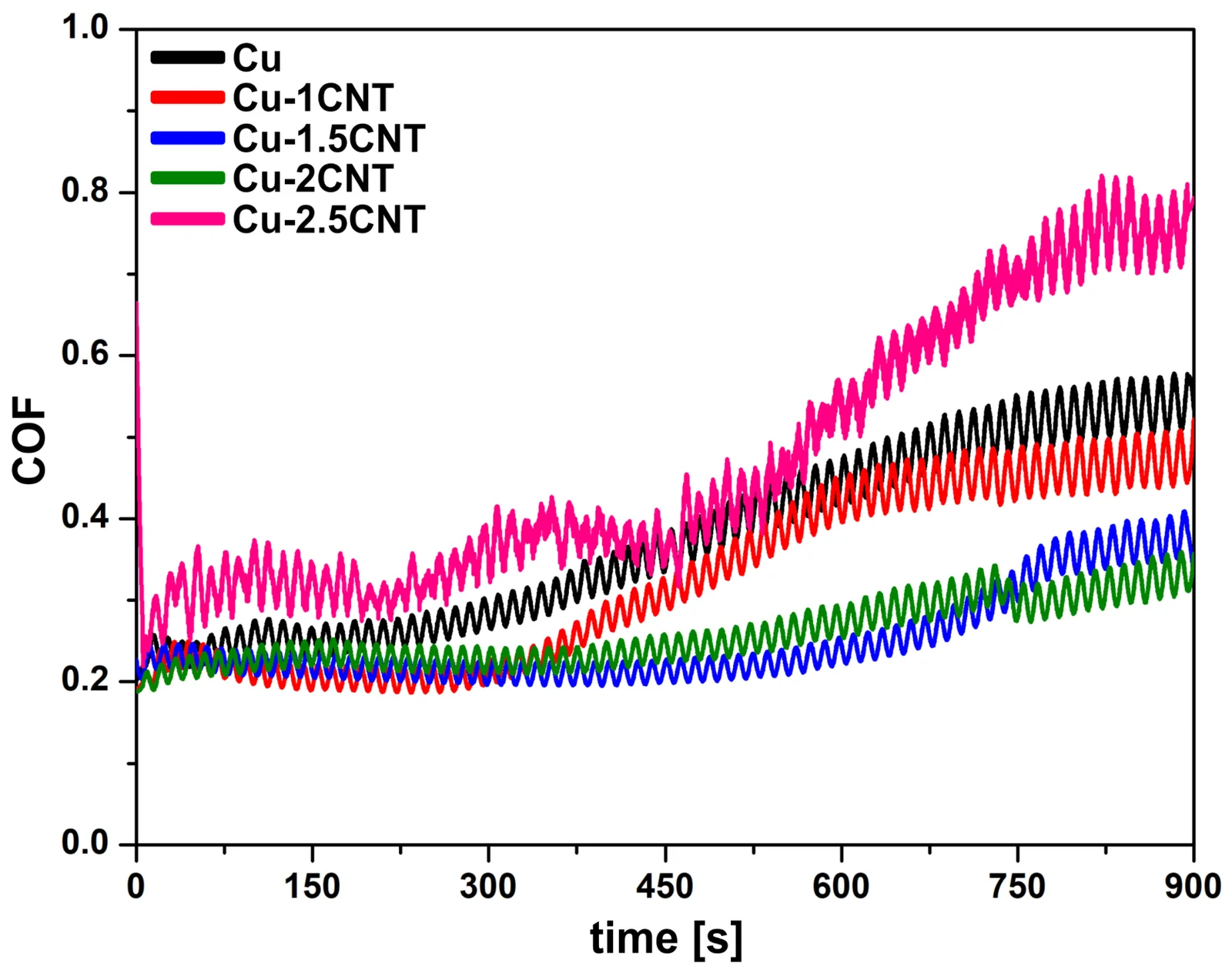
Evolution of COF as a function of sliding time for unreinforced and Cu-CNT composites.

The representative curves of the evolution of Coefficient of Friction (COF) as a function of sliding time for different unreinforced and Cu-CNT composites are presented in Figure 7.

For all groups of samples, the COF curves display a typical two-stage behaviour, where an initial low-friction regime is followed by a transition to a higher-friction regime as sliding progresses.

Overall, both unreinforced and Cu-1CNT exhibit a similar evolution of the COF throughout the test, characterized by an initial low-friction regime followed by a transition to a higher-friction steady state. During the initial low-friction stage (0–300 s), unreinforced Cu sustains a relatively low COF of c.a. 0.25. After this, a steady increase in COF is observed between 300 s and 650 s, eventually stabilizing at a higher COF of approximately 0.53, with small oscillations between 0.48 and 0.58 towards the end of the test. Through the incorporation of 1 wt.% CNT, the initial COF decreases to c.a. 0.21 and the transition to a higher-friction regime is delayed to around 330 s, before eventually stabilizing at approximately 0.47 between 600 s and 900 s.

Increasing the reinforcement fraction to 1.5 wt.% and 2.0 wt.% significantly enhances the friction response of the composites. In particular, the Cu-1.5CNT composite shows the most prolonged low-friction stage, retaining a stable COF of c.a. 0.21 up to around 550 s, after which a gradual increase up to approximately 0.38 at 900 s is observed, corresponding to a 28% reduction compared with unreinforced Cu. Similarly, the Cu-2CNT sample exhibits a low COF up until around 450 s. This period is followed by a slight increase to c.a. 0.31 at 730 s, resulting in a crossover where the Cu-2CNT composite shows a slight drop in friction and consequently leads to the lowest COF by the end of the sliding time (c.a. 0.33 at 900 s) among all tested samples, which represents a 38% reduction compared to unreinforced Cu. This improvement up to 2.0 wt.% CNT was previously observed in other studies by Taşdemir et al. [12] and Pul et al. [13], which reported optimum tribological performance at 1.5 CNT wt.% and 1–2 CNT wt.%, respectively. This increased friction response can be attributed to the combined effect of lubrication and strengthening, as CNTs reduce direct Cu/counter-body contact, stabilize friction, and often generate carbon-rich interfacial tribofilms, while also refining grains, increasing hardness, and suppressing plastic flow when dispersion and bonding are ideal [12,13].

In contrast, increasing the CNT content to 2.5 wt.% results in a worse tribological performance. After a sharp initial running-in spike in COF, the Cu-2.5CNT sample displays a high and unstable COF (0.30–0.42) during the first 450 s. Following this, the COF constantly increases, reaching approximately 0.76 by 900 s, with fluctuations varying between 0.70 and 0.82. This transition indicates that 2.5 wt.% CNT exceeds the dispersion threshold of the Cu matrix for the employed processing route. The worse friction behaviour at higher CNT content may result from increased CNT agglomeration and consequent porosity, grain-boundary congestion, or weakly bonded carbon-rich regions that can promote crack initiation or act as third-body abrasives [12].

**Figure 8:**
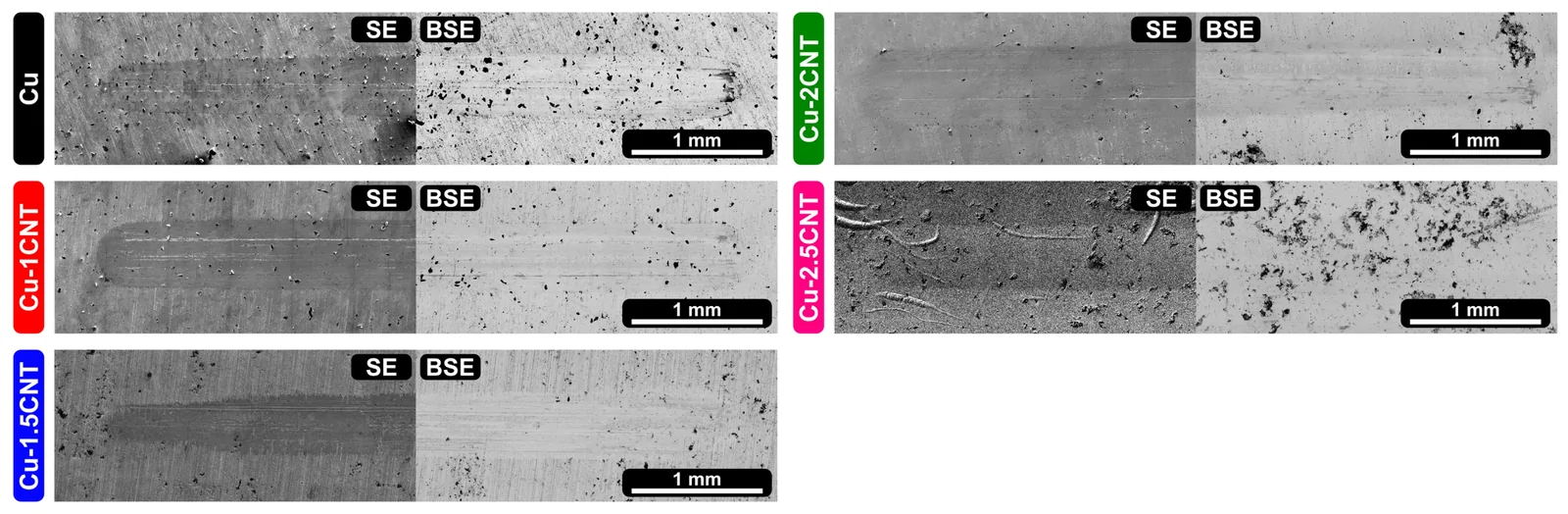
SEM-SE and SEM-BSE images of the wear tracks formed on unreinforced Cu and its nanocomposite after sliding against Al_2_O_3_ counter-bodies.

SEM-SE and SEM-BSE images of representative wear tracks formed on pure Cu and Cu–CNT nanocomposite surfaces after sliding against Al_2_O_3_ counter-bodies are presented in Figure 8.

The unreinforced Cu and Cu–1CNT samples exhibit relatively similar wear track widths. However, increasing the CNT content up to 1.5 wt.% resulted in the lowest width amongst the tested conditions. A further increase to 2.0 wt.% CNT results in the widest wear track, with the width decreasing again for the Cu–2.5CNT sample. In the case of this sample, several dark regions are visible within the wear track, possibly corresponding to CNT agglomerates and/or porosity. These features potentially contribute to the higher COF of Cu–2.5CNT compared with the other conditions, by increasing local surface heterogeneity and roughness, which can promote irregular sliding contact.

**Figure 9:**
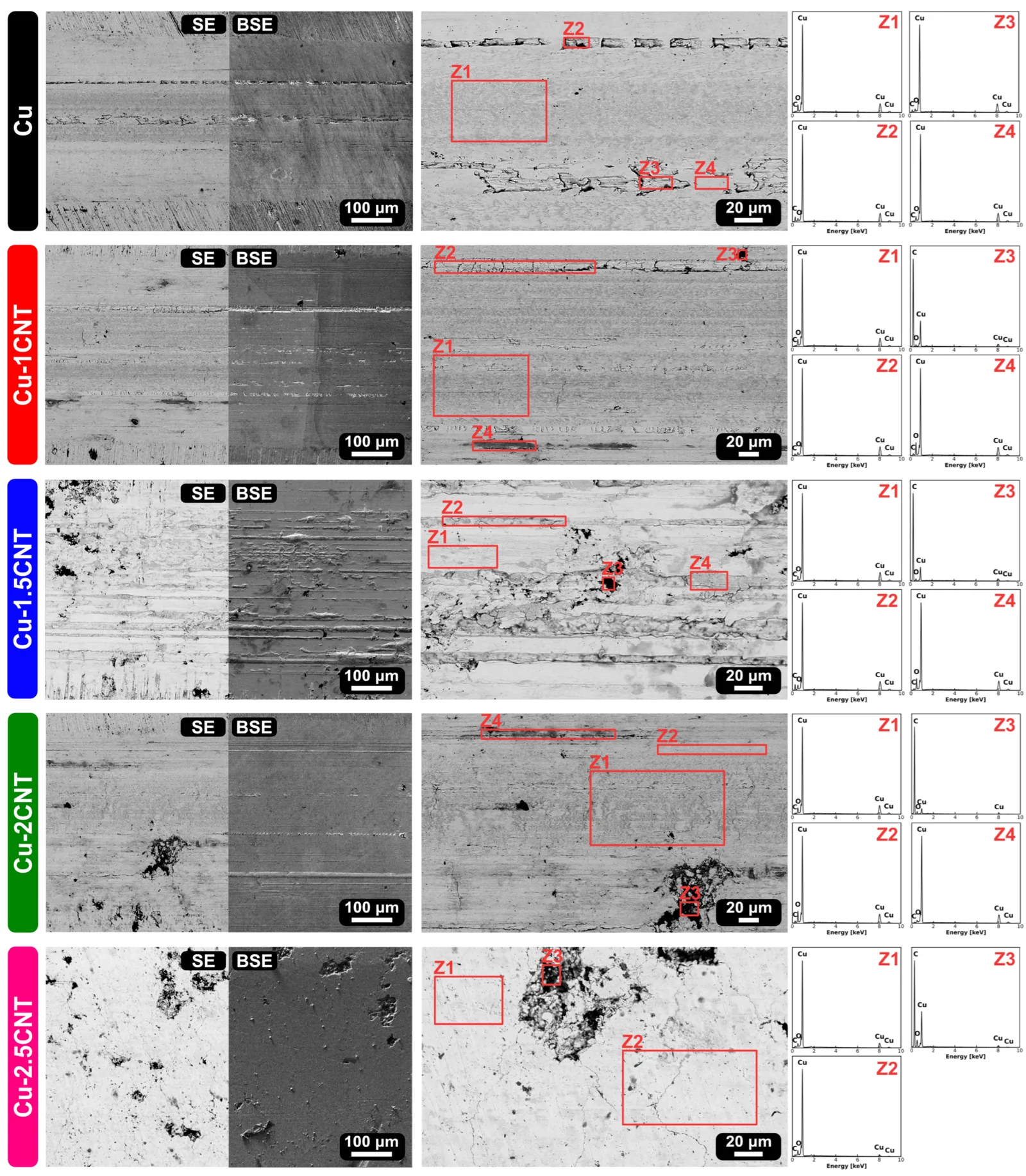
Low-magnification SEM-SE and SEM-BSE images and high-magnification SEM-BSE images of the wear tracks formed on pure Cu and Cu–CNT nanocomposite surfaces after sliding against Al_2_O_3_ counter-bodies, together with localized EDS spectra.

Higher magnification SEM micrographs together with localized EDS analysis of the wear tracks formed on unreinforced Cu and Cu–CNT nanocomposites are exhibited in Figure 9.

The unreinforced Cu sample showed pronounced parallel grooves aligned with the sliding direction, where discontinuous ploughing and delamination marks are observed along the track in regions Z2 and Z3. This morphology can indicate that the wear debris formed might act as third-body abrasion particles, which is consistent with the increasing COF and high fluctuations observed on COF evolution curves of Figure 7.

In the case of the Cu-1CNT samples, the wear track is relatively smoother, and the grooves become shallower in comparison to unreinforced Cu. The cracking of the surface is mainly confined in narrow bands (Z2), instead of the extensive and discontinuous delaminated areas observed for the unreinforced Cu sample. The dark and localized bands marked in region Z4 exhibit a strong O peak, together with high Cu signals, which could indicate that oxidation of the worn surface took place and oxidized patches were formed in the wear track. In zone Z3, reduced Cu signals with a strong C peak indicate the presence of a CNT agglomerate and/or porosity formed due to poor dispersion or low densification, which become more prominent as CNT content increases for the Cu-1.5CNT (Z3), Cu-2CNT (Z3) and Cu-2.5CNT (Z3) samples.

Increasing the CNT content to 1.5 wt.% resulted in a wear track containing elongated O-rich regions aligned with the sliding direction, indicating the development of a discontinuous oxide-rich tribolayer during sliding. In contrast, the Cu-2CNT sample showed a reduced prominence of these O-rich areas, while the surrounding matrix remains comparatively smooth.

The wear track generated on the Cu-2.5CNT sample showed the most distinct morphology, containing large CNT agglomeration/porosity areas, whereas the Cu-rich matrix of regions Z1 and Z2 exhibits fine microcracks. These can result from the presence of CNT agglomeration and porosity, which can act as stress concentrators and crack initiation sites that might produce third-body particles and increase abrasion. In addition, the presence of microcracks can indicate that a more severe fatigue-driven wear mechanism starts to take place. This further indicates a reduced load-bearing capacity of the matrix, possibly associated with weaker interfacial bonding and lower relative density as the CNT content increases beyond 2 wt.%. Moreover, porosity may act as a wear-debris reservoir, mitigating surface damage while simultaneously promoting debris accumulation and increased interfacial friction, ultimately resulting in poorer tribological performance and higher COF values.

**Figure 10:**
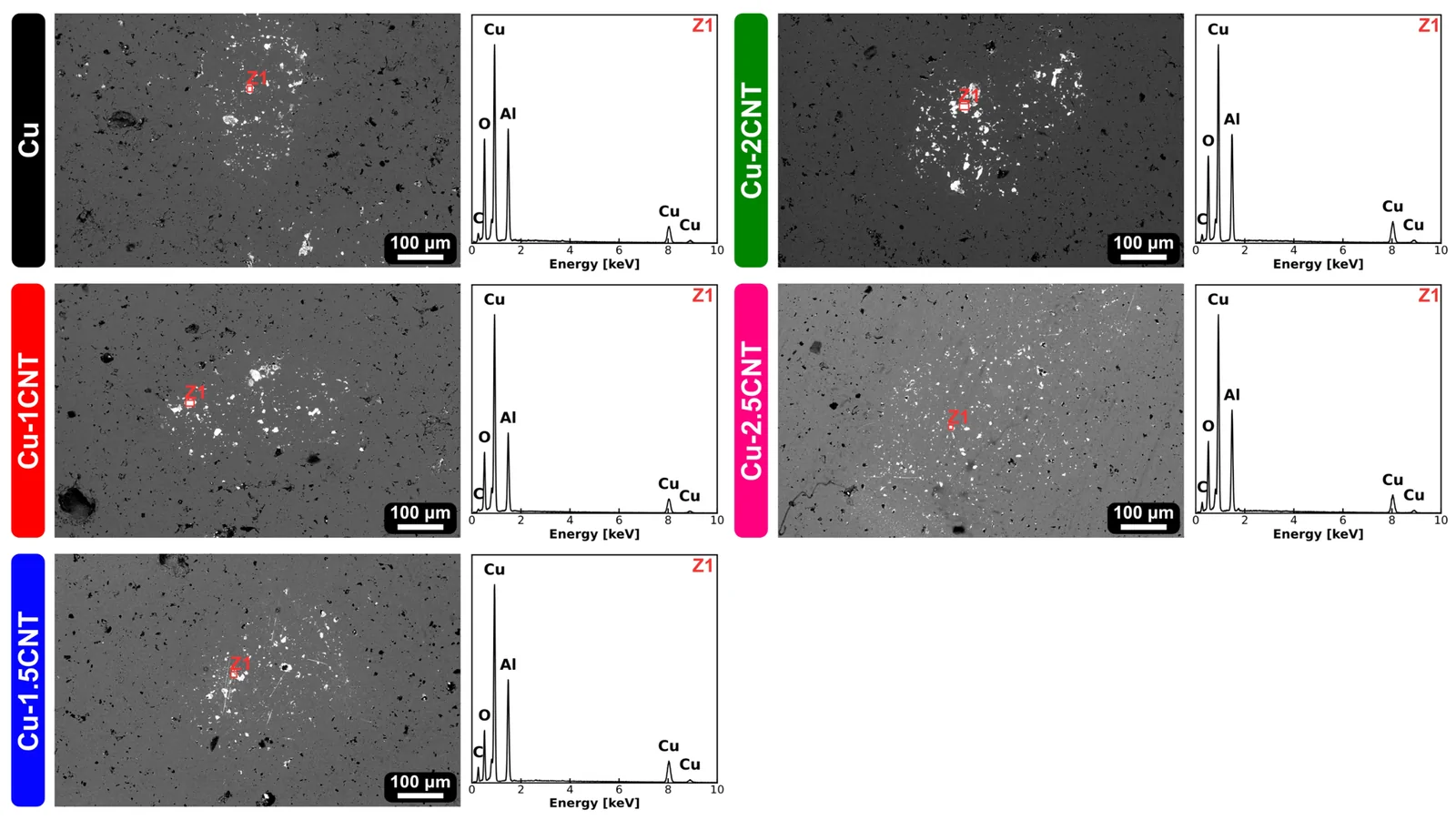
SEM-BSE images of the wear marks on the Al_2_O_3_ counter-bodies after sliding against unreinforced Cu and Cu–CNT nanocomposites, together with the corresponding localized EDS spectra.

Figure 10 exhibits the worn surfaces of the alumina counter-bodies analysed by SEM and EDS, after the wear tests. In all SEM-BSE images, the dark contrast matrix corresponds to the Al_2_O_3_ ball, while the bright contrast features arise from the material transferred from the softer unreinforced Cu or Cu matrix from Cu-CNT samples during sliding.

EDS analyses (Z1) performed directly on the bright adhered patches confirm the presence of transferred Cu and the presence of C, overlaid on the Al and O signals derived from the Al_2_O_3_ counter-body.

For the unreinforced Cu sample, the counter-body exhibits a contact scar with an area of approximately 200 × 350 µm containing discontinuous patches of smeared Cu. Compared to the CNT reinforced samples, where the peak intensity of O is superior to that observed for Al, the EDS spectrum for unreinforced Cu exhibits a substantially higher Al/O peak intensity ratio. The elevated O signal can suggest the transfer not just of Cu but also from the oxidized patches formed on the wear track during the high-friction regime, where COF was approximately 0.53.

By incorporating 1 wt.% to 2 wt.% of CNTs, the contact scars on the Al_2_O_3_ balls become less dispersed, with an area of c.a. 200 × 280 µm, indicating that the CNT-reinforced matrices restrict the Cu adhesion on the counter-body once the main load was carried by the CNT reinforcements. Additionally, the EDS spectra for Cu-1.5CNT and Cu-2CNT display a reduced Al/O ratio, together with a higher C peak, which can result from the transfer of a C-rich lubricating film. In particular, the Cu-1.5CNT sample presents the smallest and less smeared contact area with fine and dispersed Cu transferred particles, consistent with the low-friction plateau up to 550 s, while the Cu-2CNT sample showed a slightly denser transfer morphology. In contrast, the Al_2_O_3_ counter-body of the Cu-2.5CNT sample exhibits a more elongated contact scar area, with an area of c.a. 450–550 µm, despite displaying sparser and less compacted adhesion patches.

The high electrical conductivity of Cu is one of its most important functional properties, and the incorporation of CNTs is intended to enhance the mechanical and tribological performance of the material while preserving its excellent electrical transport characteristics. To assess this aspect, electrical resistivity measurements were attempted at room temperature using the four-point probe method, which was selected to minimize contact-resistance effects and provide reliable electrical characterization. However, due to the exceptionally high conductivity of both the unreinforced Cu and the Cu-CNT composites, the measured resistivity values fell below the effective detection range of the equipment, preventing a reliable quantification under the adopted experimental conditions. Although these results do not allow a direct comparison of conductivity values, they suggest that the incorporation of CNTs did not lead to a substantial reduction in the electrical conductivity of the Cu matrix, as both materials exhibited conductivity levels beyond the measurement capability of the system. Therefore, while electrical conductivity remains an important consideration for these materials, a quantitative assessment would require more sensitive low-resistance measurement techniques or specialized conductivity analyzers capable of accurately characterizing highly conductive materials.

## 4. Conclusions

The present work investigated the influence of CNT content on the microstructure, hardness, wear behaviour, and electrical conductivity of Cu-CNT nanocomposites produced by powder metallurgy. The results demonstrate that the reinforcement content plays a critical role in determining the balance between microstructural homogeneity and functional performance.

SEM observations revealed that the effectiveness of CNT reinforcement strongly depends on its dispersion within the copper matrix. The Cu-1CNT composite exhibited the most homogeneous microstructure, with a limited amount of porosity and CNT agglomerates, whereas higher CNT contents led to a progressive increase in microstructural heterogeneity. The combined area fraction of pores and CNT agglomerates increased from 1.95% for Cu-1CNT to 6.29% for Cu-2.5CNT, indicating that the dispersion capability of the selected processing route becomes increasingly limited at higher reinforcement contents. These microstructural defects negatively affected densification and reduced the effectiveness of load transfer between the matrix and the reinforcement. Hardness measurements indicated that the Cu-1CNT composite exhibited the highest hardness value (83 ± 6 HV0.01), representing a significant improvement compared with unreinforced Cu (73 ± 4 HV0.01). This enhancement was associated with the combined effects of grain refinement, increased interface density, and local strain fields generated by the CNTs. For higher CNT contents, the increasing fraction of agglomerates and porosity counteracted the reinforcement effect, resulting in a progressive reduction in hardness.

XRD analysis confirmed the preservation of the FCC copper structure after processing and showed no evidence of carbide formation or detrimental chemical reactions between Cu and CNTs. The increased peak broadening observed in CNT-containing composites indicated the development of local lattice distortions and microstrain associated with the presence of CNT-matrix interfaces. EBSD analysis further demonstrated that CNT addition promoted moderate grain refinement, reducing the average grain size from 1.34 ± 0.9 μm to 1.18 ± 0.7 μm, while also increasing local misorientation and the fraction of low-angle grain boundaries. In addition, the maximum texture intensity decreased from 2.79 to 2.49 MUD, suggesting a slight randomization of grain orientations and a modification of the recrystallization behaviour of the copper matrix.

The incorporation of CNTs significantly influenced the tribological behavior of the Cu matrix. At low to intermediate CNT contents, the composites exhibited reduced friction and improved wear resistance, which can be attributed to the reinforcing effect of CNTs and the formation of protective tribolayers during sliding. However, increasing the CNT content beyond the optimum level resulted in a deterioration of tribological performance, evidenced by higher COF values and increased wear. This behavior is likely associated with reduced matrix load-bearing capacity, weaker Cu-CNT interfacial bonding, and increased porosity. While the pores may act as wear-debris reservoirs and mitigate severe surface damage, they can also promote debris accumulation and compaction at the sliding interface, leading to increased friction. Therefore, an optimum CNT content exists at which the balance between reinforcement, tribolayer formation, and densification provides the best wear performance.

Overall, the results demonstrate that an optimum CNT content exists for Cu-CNT nanocomposites produced by the present powder metallurgy route. While 1 wt.% CNT provided the best combination of microstructural homogeneity and hardness, CNT contents between 1.5 and 2 wt.% produced the most favorable tribological performance. Above this range, the detrimental effects of CNT agglomeration and porosity outweigh the potential benefits of reinforcement. Therefore, the successful development of multifunctional Cu-CNT nanocomposites depends primarily on achieving an adequate balance between CNT content, reinforcement dispersion, and microstructural integrity, enabling significant improvements in wear resistance while preserving the excellent electrical conductivity of copper.

## Figures and Tables

**Table 1:** Evolution of hardness and porosity and/or area fraction of CNT agglomerates as a function of CNT content.

Sample	Hardness (HV0.01)	% Pores and CNT Agglomerates
Cu	73 ± 4	0.61
Cu-1CNT	83 ± 6	1.95
Cu-1.5CNT	64 ± 7	3.28
Cu-2CNT	60 ± 5	3.46
Cu-2.5CNT	52 ± 5	6.29

**Table 2:** Position (2θ) and FWHM of the main diffraction peaks identified in pure Cu and Cu-CNT nanocomposites.

Sample	Phase	2θ (°)	FWHM (°)
**Cu**	Cu_2_O	38.8104	0.1574
	Cu(111)	43.1966	0.2755
	Cu_2_O	46.0299	0.1181
	Cu(200)	50.3123	0.2362
	Cu(220)	73.9552	0.2755
	Cu_2_O	89.7606	0.1920
	Cu_2_O	90.0824	0.1920
	Cu(222)	94.9792	0.2400
**Cu-1CNT**	Cu_2_O	38.9203	0.1574
	Cu(111)	43.2608	0.2362
	Cu_2_O	45.3484	0.2362
	Cu(200)	50.3705	0.3149
	Cu(220)	74.0193	0.1968
	Cu_2_O	89.8248	0.2400
	Cu_2_O	90.1435	0.1920
	Cu(222)	95.0219	0.1920
**Cu-2CNT**	Cu_2_O	36.3996	0.3149
	Cu_2_O	38.9498	0.2362
	Cu_2_O	41.9846	0.3936
	Cu(111)	43.3057	0.2362
	Cu(200)	50.4131	0.2755
	Cu_2_O	61.5507	0.6298
	Cu(220)	74.0495	0.1968
	Cu_2_O	89.8561	0.1968
	Cu(222)	95.0474	0.1920

**Table 3:** Summary of the main EBSD parameters obtained for the Cu matrix and Cu-1CNT.

Sample	Grain Size	Grain Boundaries Character	KAM Angle
**Cu**	1.34 ± 0.9	2° < angle < 15° = 0.079	0.55
		CSL = 0.56	
		Angle > 15° = 0.37	
**Cu-1CNT**	1.18 ± 0.7	2° < angle < 15° = 0.090	0.58
		CSL = 0.53	
		Angle > 15° = 0.38	

## Data Availability

The authors confirm that the data supporting the findings of this study are available within the article.

## References

[1] Ren X., Chang Y., Ge C. (2024). State-of-the-Art Carbon-Nanotubes-Reinforced Copper-Based Composites: The Interface Design of CNTs and Cu Matrix. Int. J. Mol. Sci..

[2] Xiu Z., Sun J., Li X., Chen Y., Yan Y., Shao P., Li H., Ju B., Yang W., Chen G. (2024). Study on Mechanical and Electrical Properties of High Content CNTs/Cu Composites. Materials.

[3] Jayathilaka W.A.D.M., Chinnappan A., Ramakrishna S. (2017). A Review of Properties Influencing the Conductivity of CNT/Cu Composites and Their Applications in Wearable/Flexible Electronics. J. Mater. Chem. C.

[4] Iijima S. (1991). Helical Microtubules of Graphitic Carbon. Nature.

[5] Carneiro Í., Simões S. (2021). Strengthening Mechanisms in Carbon Nanotube Reinforced Metal Matrix Composites: A Review. Metals.

[6] Kim K.T., Eckert J., Menzel S.B., Gemming T., Hong S.H. (2008). Grain Refinement Assisted Strengthening of Carbon Nanotube Reinforced Copper Matrix Nanocomposites. Appl. Phys. Lett..

[7] Fu S., Chen X., Liu P., Cui H., Zhou H., Ma F., Li W. (2022). Tribological Properties and Electrical Conductivity of Carbon Nanotube-Reinforced Copper Matrix Composites. J. Mater. Eng. Perform..

[8] Daoush W.M. (2008). Processing and Characterization of CNT/Cu Nanocomposites by Powder Technology. Powder Metall. Met. Ceram..

[9] Bakshi S.R., Lahiri D., Agarwal A. (2010). Carbon Nanotube Reinforced Metal Matrix Composites—A Review. Int. Mater. Rev..

[10] Ya B., Xu Y., Meng L., Zhou B., Zhao J., Chen X., Zhang X. (2022). Fabrication of Copper Matrix Composites Reinforced with Carbon Nanotubes Using an Innovational Self-Reduction Molecular-Level-Mixing Method. Materials.

[11] Humphreys F.J., Hatherly M. (2004). Recrystallization and Related Annealing Phenomena.

[12] Taşdemir F., Özyürek D., Yıldırım M. (2019). Effect of Carbon Nanotube Content on the Wear Behaviours of Cu-CNT Composites Produced by Powder Metallurgy Method. Acta Phys. Pol. A.

[13] Pul M., Yilmazel R., Erten M.Y., Küçüktürk G., Kaya D., Salunkhe S., Zümrüt Y., Čep R., Nasr E.A.A. (2023). Investigation of the Effect of Lubricant Properties of Carbon Nanomaterial in Cu/MWCNT Composites on Wear. Front. Mater..

[14] Carneiro Í., Simões S. (2020). Effect of Morphology and Structure of MWCNTs on Metal Matrix Nanocomposites. Materials.

[15] Carneiro Í., Monteiro B., Ribeiro B., Fernandes J.V., Simões S. (2023). Production and Characterization of Cu/CNT Nanocomposites. Appl. Sci..

[16] Carneiro Í., Fernandes J.V., Simões S. (2021). Investigation on the Strengthening Mechanisms of Nickel Matrix Nanocomposites. Nanomaterials.

[17] Monteiro B., Simões S. (2024). Production and Characterization of Hybrid Al6061 Nanocomposites. Metals.

[18] Yan Y.F., Kou S.Q., Yang H.Y., Shu S.L., Qiu F., Jiang Q.C., Zhang L.C. (2023). Ceramic Particles Reinforced Copper Matrix Composites Manufactured by Advanced Powder Metallurgy: Preparation, Performance, and Mechanisms. Int. J. Extrem. Manuf..

[19] Kumar N., Bharti A., Dixit M., Nigam A. (2020). Effect of Powder Metallurgy Process and its Parameters on the Mechanical and Electrical Properties of Copper-Based Materials: Literature Review. Powder Metall. Met. Ceram..

[20] Borges J., Martin J., Barradas N.P., Alves E., Eyidi D., Beaufort M.F., Riviere J.P., Vaz F., Marques L. (2012). Electrical Properties of AlNxOy Thin Films Prepared by Reactive Magnetron Sputtering. Thin Solid Films.

[21] Mallikarjuna H.M., Ramesh C.S., Koppad P.G., Keshavamurthy R., Sethuram D. (2017). Nanoindentation and Wear Behaviour of Copper-Based Hybrid Composites Reinforced with SiC and MWCNTs Synthesized by Spark Plasma Sintering. Vacuum.

[22] Huang Z., Zheng Z., Zhao S., Dong S., Luo P., Chen L. (2017). Copper Matrix Composites Reinforced by Aligned Carbon Nanotubes: Mechanical and Tribological Properties. Mater. Des..

[23] Lin C.B., Chang Z.C., Tung Y.H., Ko Y.Y. (2011). Manufacturing and Tribological Properties of Copper Matrix/Carbon Nanotubes Composites. Wear.

[24] Kim K.T., Cha S.I., Hong S.H. (2007). Hardness and Wear Resistance of Carbon Nanotube Reinforced Cu Matrix Nanocomposites. Mater. Sci. Eng. A.

[25] Rajkumar K., Aravindan S. (2011). Tribological Studies on Microwave Sintered Copper-Carbon Nanotube Composites. Wear.

[26] Xiao Y., Zhang Z., Yao P., Fan K., Zhou H., Gong T., Zhao L., Deng M. (2018). Mechanical and Tribological Behaviors of Copper Metal Matrix Composites for Brake Pads Used in High-Speed Trains. Tribol. Int..

